# Association of parental oral health literacy and child temperament with dental attendance in children with early childhood caries: a cross-sectional study

**DOI:** 10.1007/s40368-025-01083-w

**Published:** 2025-07-11

**Authors:** D. Hegde, B. S. Suprabha, R. Shenoy, A. Rao

**Affiliations:** 1 Department of Pediatric and Preventive Dentistry, Manipal College of Dental Sciences Mangalore, Manipal Academy of Higher Education, Manipal, India; 2 Department of Public Health Dentistry, Manipal College of Dental Sciences Mangalore, Manipal Academy of Higher Education, Manipal, India

**Keywords:** Dental caries, Child, Preschool, Temperament, Health literacy, Parents

## Abstract

**Purpose:**

To study the associations of parental oral health literacy (OHL) and child temperament with nonattendance during recall appointments among children with early childhood caries (ECC).

**Methods:**

This cross-sectional study involved 100 parent‒child dyads with children aged 71 months or younger affected by ECC (at least one decayed, missing or filled tooth). After providing informed consent, the accompanying parents completed a questionnaire assessing their attitudes towards oral health, barriers to dental care utilisation, OHL (Oral Health Literacy Adult Questionnaire) and their child’s temperament (Emotionality, Activity, Shyness Temperament Survey for Children). Data on dental appointment cancellations, missed appointments, or discontinuations over 6 months were collected retrospectively from patient records. Participants with ≥ 20% cancelled or missed appointments or treatment discontinuation were categorised as nonattenders; the rest were attenders. The associations between nonattendance and parental OHL, child temperament and other factors were analysed using the chi-square test, Student's t test and multiple logistic regression analysis.

**Results:**

Of the 100 child patients, 47 were attenders, and 53 were nonattenders. There was no significant difference in parental OHL levels between the attenders and nonattenders. Multiple logistic regression analysis revealed a significant association between nonattendance and activity temperament (odds ratio (OR) = 1.90, *p* = 0.019), lack of time (OR = 4.24, *p* = 0.041), and inconvenience (OR = 6.00, *p* < 0.001).

**Conclusion:**

The activity temperament of the child, parental perceptions of lack of time, and inconvenience are associated with nonattendance among children with ECC. The association of parental OHL with nonattendance for dental appointments could not be established.

## Introduction

Early childhood caries (ECC) is an oral disease that is prevalent globally and affects children ≤ 6 years of age (American Academy of Pediatric Dentistry 2024). Access to dental care and visits early in life suppresses the disease (Beil et al. 2014). Regular examination by dentists and preventive measures can decrease treatment costs, improve oral health-related quality of life, and ensure healthy development (Cunnion et al. 2010; Beil et al. 2012).

Dental care faces a well-recognised problem of nonattendance, as in other health services (Gustafsson et al. 2018). Nonattendance refers to missing or cancelling scheduled dental appointments or discontinuing dental treatment (Gustafsson et al. 2010). A Danish study revealed that 38% of children in the 6–8-year age group had missed one or more appointments over 3–6-year intervals (Wogelius and Poulsen 2005). In contrast, another study on children between 7.5 and 18 years of age reported that 11% had cancelled appointments over 3 years (Gustafsson et al. 2010). Nonattendance increases the risk of dental caries among children and increases the cost of dental health care (Wang and Aspelund 2009). Missing or cancelling appointments prevents other patients from obtaining care, imposes added strain on the system, and extends wait times for people for appointments, affecting their quality of care (Alturbag 2024). An understanding of the factors leading to nonattendance will help in better organising provisions for dental care and planning patient recall routines (Wang and Aspelund 2009).

Health literacy is defined by Sørensen et al. (2012) as “linked to literacy and entails people’s knowledge, motivation and competences to access, understand, appraise, and apply health information to make judgments and take decisions in everyday life concerning healthcare, disease prevention and health promotion to maintain or improve quality of life during the life course”. Lower levels of health literacy can contribute to poor health outcomes (Berkman et al. 2011). The oral health literacy construct is similar to health literacy in the medical field. Oral health literacy (OHL) is described as the"degree to which individuals have the capacity to access, process and understand basic oral and craniofacial health information and services needed to make appropriate health decisions"(U.S. Department of Health and Human Services 2000). In essence, OHL is a set of skills that help individuals operate effectively in the oral healthcare environment (Firmino et al. 2018).

Parents play a central role in maintaining their child's oral health (Castilho et al. 2013). Hence, the current focus is on parental/caregiver health literacy. Low health literacy among parents is associated with poor health behaviours that affect their children's health practices, suggesting a correlation between low parental health literacy and poor child health outcomes (DeWalt and Hink 2009). The association of low parental OHL with dental caries among children has been confirmed in systematic reviews (Firmino et al. 2018; Alzahrani et al. 2024). Parents often cannot act on their children's oral health needs because of low OHL (Alzahrani et al. 2024). Parental oral health behaviours mediate the relationship between parental OHL and dental caries levels in children (Wu et al. 2024). Parental OHL can influence the frequency of a child’s tooth brushing and sugar consumption in the diet (Sistani et al. 2017; Sowmya et al. 2021), although studies on the associations between dietary frequency of sugars and OHL have yielded conflicting results (Buja et al. 2021; Alzeer et al. 2024). While parental OHL has been associated with inadequate oral hygiene measures for children, particularly a lack of help in tooth brushing and night-time feeding with bottles, the role of parental OHL in the regularity of dental visits has not been confirmed (Alzahrani et al. 2024; Wu et al. 2024). The results of studies on the association of parental OHL with the regularity of dental attendance have been conflicting because of the variations in the study populations and OHL measures used (Firmino et al. 2018; Badran et al. 2023; Alzeer et al. 2024). A recent study revealed that parents’ low OHL was associated with a low prevalence of dental visits by preschool children (Menoncin et al. 2023). Considering these findings, it was hypothesised that parental OHL may influence the nonattendance of child patients with ECC during their recall dental appointments.

Temperament refers to different but relatively stable characteristics of an individual's response to the environment (Boer and Westenberg 1994). Temperament determines an individual's reaction to a new scenario, such as how a child reacts during the first dentist appointment (Klingberg and Broberg 1998). Different dimensions of temperament include emotionality, which manifests as distress behaviour; activity, which represents the tempo and vigour of the individual and inclination towards physical activity; and shyness, which manifests as inhibition and tension behaviour during encounters with strangers (Boer and Westenberg 1994). Temperament has been shown to influence children's dental anxiety and dental behaviour (Jain et al. 2019; Paiva et al. 2023; Do et al. 2023). Children with behaviour management problems more often missed or cancelled their dental appointments because of dental fear and inability to cope with treatment (Arnrup et al. 2003). Children’s aversion to dental visits and dental anxiety can also affect the ability of parents to bring their children for dental appointments regularly (Badri et al. 2014, Carillo-Diaz et al. 2021). In an earlier study involving adolescents, an impulsive temperament was associated with nonattendance for dental treatment (Gustafsson et al. 2010). However, whether personality characteristics such as the temperament of preschool children with ECC influence nonattendance for dental treatment during recall appointments is not known.

Thus, this study aimed to examine the associations between parental oral health literacy and children's temperament with nonattendance for scheduled recall dental appointments among children with early childhood caries (ECC), using the Oral Health Literacy Adult Questionnaire and the Emotionality, Activity, Shyness Temperament Survey for Children (parental ratings), respectively.

## Methods

### Study design, setting and sampling

This cross-sectional study was conducted at the dental clinic of a dental school's Department of Paediatric and Preventive Dentistry at Mangalore, a city in southern India. The dental school spans about 114,000 square feet and houses clinics with 311 dental chairs. It admits 100 undergraduate and 31 postgraduate students annually. On average, 446 patients are treated daily across nine departmental clinics, including the Department of Pediatric and Preventive Dentistry. While reporting this study, the STROBE guidelines (Vandenbroucke et al. 2014) were followed. A convenience sample of parent‒child dyads was selected from those attending the dental clinic for treatment. The participants were recruited from May 2022 to October 2022.

*Inclusion Criteria*: Children aged 71 months or younger with one or more decayed teeth (noncavitated or cavitated lesions), missing teeth (due to caries), or with filled with tooth surfaces in any primary tooth (as per the definition of ECC) (American Academy of Pediatric Dentistry, 2024), who were accompanied by any one of their parents or guardians, and who had undergone treatments in the dental clinic with at least two earlier dental appointments within six months before the date of their inclusion in the study. The participants were included if the accompanying parent knew how to read and write the English language.

*Exclusion criteria*: Children with medical and developmental disorders, communicative disorders and special health care needs or whose parents/guardians did not consent to participate were excluded from the study.

*Sample size*: The software G Power 3.1.2. [G*Power 3.1.2 software (Heinrich-Heine Universität Düsseldorf), Germany (Free version)] was used to calculate the sample size. The sample size was determined, assuming 52% nonattendance among child patients (Bhatia et al. 2018). The sample size was calculated as 100, with a critical chi-square value of 11.07 at a 95% confidence interval and 80% power, assuming a 4% difference between attenders and nonattenders.

### Ethical considerations

The study was conducted with prior permission from the Institutional Ethics Committee (Ref. no. 21017). The parents of the children eligible to participate in the study were given a participant information sheet, and they signed a written informed consent form before being included in the study. Parents were informed that participation in the study was voluntary and that nonparticipation would not compromise the subsequent treatment of their child in any manner.

### Procedure

The participating parent/guardian was provided with a questionnaire that included questions about the age and sex of the child patient and the participating parent/guardian, socioeconomic status (SES), reason for first dental visit (complaint of pain or not), past dental experience of the child (Likert scale with scores ranging from 1 to 5: very unpleasant = 1, unpleasant = 2, neutral = 3, pleasant = 4, very pleasant = 5), parental attitudes towards the oral health and general health of their child, and barriers to dental care utilisation for their children (Gao et al. 2020). The parents’ attitudes towards their child's oral and general health were evaluated with a single question each: “How do you evaluate the oral health of your child?” and “How do you evaluate the general health of your child?”, for which the response was obtained on a Likert scale. Furthermore, attitudes towards the importance of oral health and prevention of dental caries, as well as the importance of protecting children's teeth from dental caries, were included in the questionnaire, for which responses were obtained on a Likert scale. The Likert scale scores for all parental attitudes ranged from 1 to 5; good = 5, fair = 4, moderate = 3, bad = 2, worst = 1). Data on barriers to regular attendance, such as lack of perceived need for treatment, awareness about dental disease in their children, fear of pain during treatment, cost of dental treatment, difficulty in communicating with the dentist, difficulty in accessing dental care, breaks in school attendance, lack of time and inconvenience in bringing children for multiple dental appointments, were also obtained. Data on the above aspects were collected, as these factors could influence nonattendance, in accordance with earlier literature (Skaret et al. 2000; Wang and Aspelund 2009; Gao et al. 2020). The questionnaire was validated by two subject experts for content and sequence validity. The test–retest reliability of the questionnaire was assessed prior to the study by administering it to 10 parents who were not a part of the study sample twice at an interval of two weeks, and Cohen's kappa (κ) values were obtained. The Kuppuswamy scale was utilised to assess SES (Saleem and Jan 2021).

### Assessment of parental oral health literacy

The Oral Health Literacy Adult Questionnaire (OHL-AQ) (Naghibi Sistani et al. 2014) was utilised for the parental OHL assessment. This tool includes 14 questions (17 responses) in four sections: reading comprehension, numeracy, listening, and decision-making. The reading comprehension test consisted of three fill-in-the-blanks on oral health knowledge, with five options each, including a “do not know” option. The numeracy section had two prescription cards on amoxicillin and sodium fluoride with instructions and two questions per card. The participants were instructed to read and write or pick up responses regarding the prescription. For the listening section, post-extraction instructions were read aloud twice by a single interviewer. The participants were expected to listen to the interviewer and answer two questions. The last decision-making component had five questions about typical oral health conditions, with four options each.

The interviewer only read out the instructions for the listening section. No help was provided to the parent in reading, understanding the conceptual meaning of the items and answering the questions. The principal investigator checked the questionnaire for unanswered items and requested the parents to answer them. Each objective question had a single right answer, and the total number of correct answers was recorded for each participant. Each correct response was given a score of 1, and the total score was calculated, which ranged from 0 to 17. OHL scores were classified into three categories: inadequate, marginal, and adequate, with scores ranging from 0 to 9, 10–11, and 12–17, respectively.

### Assessment of the child’s temperament

The child's temperament was measured via the EAS (Emotionality, Activity, Shyness) Temperament Survey for Children (parental ratings), which consists of 15 items, 5 for each of the three temperaments. The minimum and maximum scores for each temperament type were 5 and 25, respectively. Each child received a mean score for each temperament type (Boer and Westenberg 1994). The accompanying parent completed the EAS questionnaire.

The reliability of the OHL-AQ and EAS (parental ratings) scales was examined before the study began by requesting parents of 10 children excluded from the study's final sample to answer the questionnaire at 2-week intervals. Cronbach's α values were calculated to assess the stability of the responses over time. The test‒retest reliability assessment was conducted to ensure that parents understood and consistently responded to the questionnaire. Minor adjustments in language were made to two items for better comprehension of the EAS questionnaire, based on an earlier study where excellent consistency was obtained for the EAS questionnaire with language modifications (Jain et al. 2019). Specifically, the phrases'is always on the go'and'is off and running as soon as he wakes up in the morning'were changed to'is active or very busy'and'makes a good start and progresses well with his activities as soon as he wakes up in the morning'. The principal investigator reviewed each completed questionnaire to ensure that the parents answered all the questions. If any responses were missing, the investigator requested that the parents complete them. As a result, no questionnaires were returned with unanswered questions.

Data on dental appointment cancellations, missed appointments, or discontinuations over the 6-month retrospective period were collected from patient records. A missed appointment was defined as a child patient failing to appear or cancel the appointment less than 24 h before the scheduled appointment. When there were no shows for two consecutive appointments, it was determined as'discontinued'. The time interval between the appointments was also recorded. During each of the previous appointments, as part of the routine treatment protocol in the department, patients were given appointment cards indicating the date and time of the next appointment. They were given one reminder via telephone call one day before the appointment. Participants with ≥ 20% cancelled or missed appointments or discontinuation of the treatment during the retrospective 6-month period were categorised as nonattenders, and the remaining participants were categorised as attenders (Gustafsson et al. 2010). The calculations were performed as a percentage of the total number of appointments received by the child patient during the retrospective 6-month period.

### Statistical analysis

IBM SPSS software, version 29, was used for data analysis. Descriptive statistics were calculated. Statistical analysis consisted of an independent Student's t test (for mean values) and a chi-square test (for categorical data). Multiple logistic regression was applied to assess the associations of nonattendance (dependent variables: attenders and nonattenders) with independent variables, such as parental oral health literacy, child temperament, and other covariates that can influence dental nonattendance, such as demographic variables, time intervals between appointments, and reasons provided by parents for nonattendance of their child. For all the tests, the significance level was 0.05 (*p* < 0.05).

## Results

**Fig. 1:**
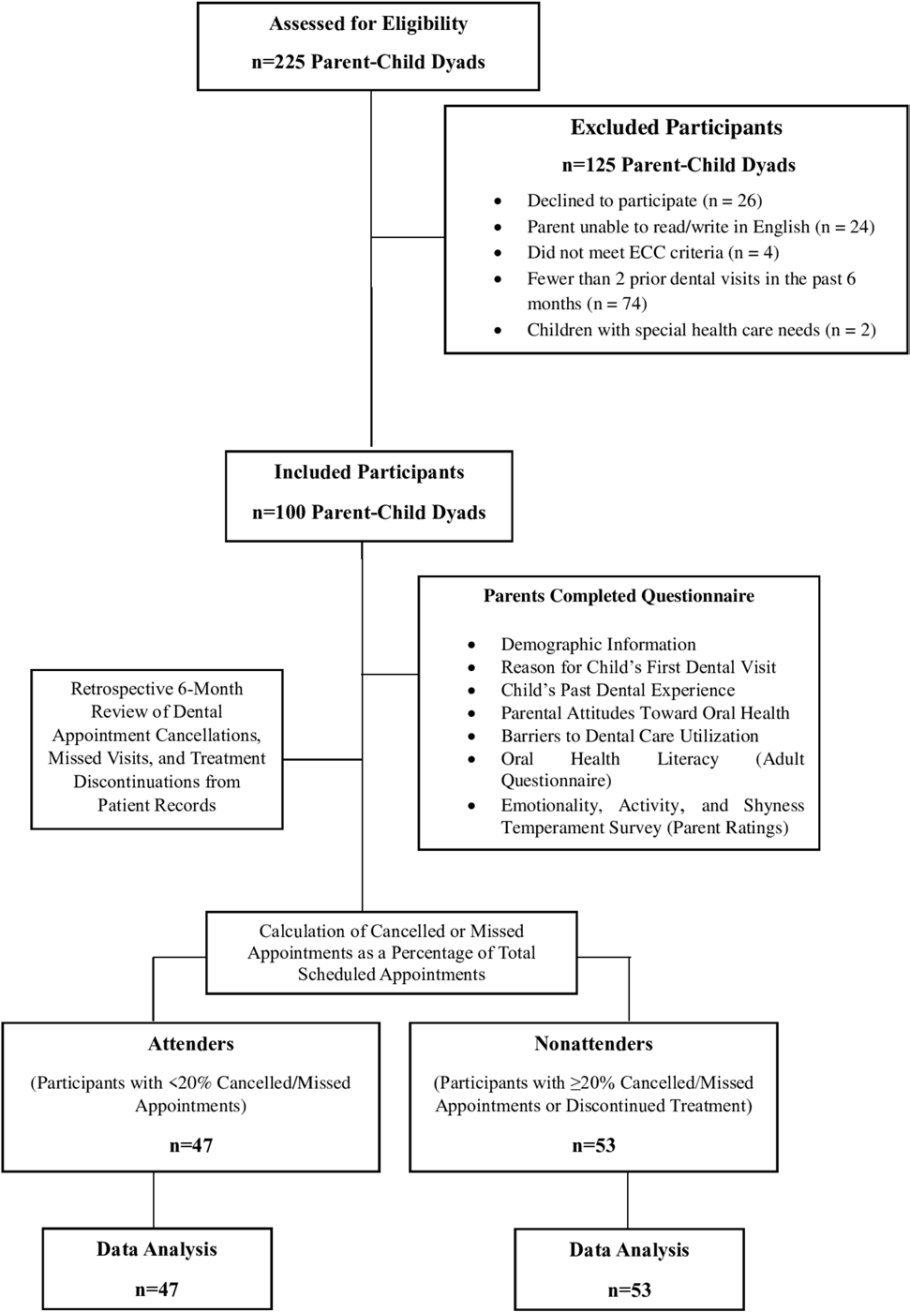
Flow chart depicting the flow of participants in the study

A total of 225 patients ≤ 71 months of age were screened for eligibility. The parent‒child dyads were not included in the study if they did not fulfil any of the inclusion criteria and were excluded by the exclusion criteria. One hundred children with ECC, accompanied by any of their parents or guardians who met the inclusion criteria and provided informed consent to answer the questionnaire participated in the study. The flow of participants into the study is illustrated in Fig. 1. The mean age of the participating parents/guardians was 35.65 ± 5.19 years, and the mean age of their children who underwent treatment for ECC was 4.76 ± 0.64 years. The participating parents or guardians included 19 fathers, two grandfathers and 79 mothers. Among the children, 49 were male, and 51 were female. The descriptive statistics of the attenders and nonattenders are provided in Tables 1 and 2. The sociodemographic factors of the participating parent‒child dyads did not differ significantly. All the parents belonged to either upper-middle or upper-lower SES categories.

**Table 1:** Comparison of the mean age of the child and parent between attenders and nonattenders

Variable	Attenders		Nonattenders		*t* value	*P* value
	Mean ± SD	Min- Max	Mean ± SD	Min–Max		
Age child (years)	4.68 ± 0.73	4–6	4.83 ± 0.55	3–6	1.17	0.253
Age parent (years)	35.66 ± 5.27	30–40	35.70 ± 5.18	28–65	1.15	0.971

**Table 2:** Comparison of the sex of the child and parent and the socioeconomic status of the attenders and nonattenders

Variable		Attenders	Nonattenders	*χ*2	*P* value
		*N* (%)	*N* (%)		
Sex (child)	Male	21 (44.7)	28 (52.8)	0.662	0.416
	Female	26 (55.3)	25 (47.2)		
Sex (parent)	Male	7 (14.9)	14 (26.4)	1.993	0.158
	Female	40 (85.1)	39 (73.6)		
Socioeconomic status	Upper middle	43 (91.5)	48 (90.6)	0.026	0.872
	Upper lower	4 (8.5)	5 (9.4)		

Among the 100 child patients, 47 were classified as attenders and 53 as nonattenders based on ≥ 20% cancellations, missed appointments and treatment discontinuation criteria. There were two appointment cancellations by the parents, two discontinued treatments, 66 patients had at least one missed appointment, and the remaining 30 did not miss a single appointment. The children had received a minimum of two appointments and a maximum of eight appointments during the 6-month retrospective period. The frequencies of patients who received third, fourth, fifth, sixth, seventh and eighth appointments were 85, 57, 38, 28, 22 and 11, respectively, based on their treatment needs. There was a statistically significant difference in the number of missed/cancelled appointments between attenders and nonattenders. No statistically significant difference was noted in the time interval for appointments, and the total number of appointments between attenders and nonattenders (Table 3).

**Table 3:** Frequencies of the number of missed appointments, total number of appointments per participant, and mean time interval of appointments between attenders and nonattenders

Variable		Attenders	Nonattenders	Statistical test constant	*P* value
		*N* (%)	*N* (%)		
Mean time interval between appointments		5.38 ± 3.99 (0.67–22.33)	6.78 ± 4.14^b^ (1.33–20.00)^c^	1.73^a^	0.087
Number of missed appointments	None	30 (63.8)	…	65.00^d^	< 0.001*
	One	17 (36.2)	33 (62.3)		
	Two	…	15 (28.3)		
	Three	…	5 (9.4)		
Total number of appointments for each participant	2	8 (17.0)	7 (13.2)	6.16^d^	0.414
	3	14 (29.8)	19(35.8)		
	4	4 (8.5)	10 (18.9)		
	5	8 (17.0)	4 (7.5)		
	6	3 (6.4)	1 (1.9)		
	7	6 (12.8)	5 (9.4)		
	8	4 (8.5)	7 (13.2)		

^*^p < 0.05-significant

^a^Student’s *t* test (*t* value)

^b^Mean ± standard deviation (SD)

^c^Range (Minimum–Maximum)

^d^Fisher’s exact test

The reliability of the OHL questionnaire for the study sample was good (Cronbach's *α* = 0.807). There was no significant difference in the oral health literacy levels of the participating parents/guardians between the attenders and nonattenders. Fifty participating parents/guardians had adequate oral health literacy scores, 19 scored marginally, and 31 had inadequate oral health literacy scores. Thirty-two percent of the parents of nonattenders had inadequate OHL scores, whereas approximately 30% of the parents of attenders also had inadequate scores (Table 4).

**Table 4:** Comparison of oral health literacy (OHL) scores among attenders and nonattenders

OHL score	Attenders *N* (%)	Nonattenders *N* (%)	*χ*2	*P* value
Adequate	23 (48.9)	27(50.9)	0.304	0.859
Marginal	10 (21.3)	9 (17.0)		
Inadequate	14 (29.8)	17 (32.1)		

The Cronbach's α values of the EAS questionnaire for the emotionality, activity and shyness parameters indicated excellent response stability (Cronbach's *α* = 0.996, 0.975, and 0.971, respectively). The children's temperament was scored according to three parameters, and the mean emotionality, shyness, and activity values are provided in Table 5. There was no statistically significant difference in the mean temperament scores between the attenders and nonattenders.

**Table 5:** Comparison of the mean temperament scores among attenders and nonattenders

Temperament	Attender	Nonattenders	*t* value	*P* value
	Mean ± SD	Mean ± SD		
Emotionality	10.96 ± 3.18	11.94 ± 3.37	0.137	0.991
Activity	18.23 ± 0.98	17.79 ± 1.72	0.125	0.078
Shyness	14.81 ± 2.83	14.19 ± 3.22	0.312	0.160

The test–retest reliability of the questionnaire for the covariates assessed for their possible influence on dental attendance, such as the past dental experience of the child, parental attitudes towards oral health, and barriers to dental care utilisation, ranged from *κ* = 0.82–1, implying excellent agreement. Among the attenders, 19 (40.4%) children and 20 (51.3%) children who were nonattenders had visited other dental clinics in the past (*χ*2 = 0.08, *p* = 0.783), and all those who had visited other dental clinics in the past reported that their child's experience with dental treatment was ‘pleasant’. All parents also rated their children’s prior visits to our dental clinic as ‘pleasant’. All participants’ first visit to the dental clinic was due to tooth pain. All participants had a history of occasional pain in the past 12 months, but one participant among the nonattenders reported the pain frequency as'often'in the past 12 months.

Parents'attitudes concerning their child's general and oral health were statistically similar among attenders and nonattenders (Table 6). All parents responded that their child's oral health and general health were either good or fair, with none of the parents assessing them as bad or worse. The proportion of parents who rated their child's oral health as'good'rather than'fair'was greater among the nonattenders. However, it was not significantly greater than that of the attenders. All the participants agreed that “oral health is important in our life” and that “it is important to protect my child’s teeth from tooth decay”. None of the parents perceived that their child had no dental disease or that their child’s dental disease was not severe. They perceived the need to treat primary teeth and knew that carious primary teeth require dental treatment.

**Table 6:** Comparison of parental attitudes toward the general and oral health of their children between attenders and nonattenders

Parental Perception		Attenders *N* (%)	Nonattenders *N* (%)	*χ*2	*P* value
General Health	Good	37 (78.7)	44 (83.0)	0.299	0.585
	Fair	10 (21.3)	9 (17.0)		
Oral health	Good	18 (38.3)	28 (52.8)	2.118	0.146
	Fair	29 (61.7)	25 (47.2)		

The most common barriers to nonattendance for dental appointments were'inconvenience'and lack of time, which were significantly greater among the nonattenders. The child's poor health and family commitments were the reasons for'inconvenience'. Two parents reported forgetting the dental appointment as the reason for'inconvenience'. Reasons such as fear of contracting infectious diseases due to dental visits, lack of availability of a dentist nearby, and breaks in school attendance due to dental appointments were indicated only by a few parents. Among the other possible barriers included in the questionnaire, no parents cited difficulty communicating with the dentist, the reliability of the dentists in delivering good dental treatment, the fear of pain and the cost of dental treatment as barriers to bringing their children for dental appointments (Table 7).

**Table 7:** Comparison of the reasons provided by the parents for missing dental appointments

Reason for missed appointments	Attenders *N* (%)	Nonattenders *N* (%)	*χ*2	*P* value
Inconvenience	13 (27.7)	39 (73.6)	21.05	< 0.001*
Lack of time	11(23.4)	28 (50.9)	8.02	0.004*
Fear of infectious diseases	1 (2.1)	4 (7.5)	1.54	0.215
No dentists nearby	1 (2.1)	1 (1.9)	0.01	0.932
Break in school attendance	1 (2.1)	4 (7.5)	1.54	0.215

*P* < 0.05 = significant

Multiple logistic regression analysis with dental attendance (attenders and nonattenders) as the dependent variable and parental oral health literacy, child temperament, demographic variables, time interval between appointments, and reasons provided by parents for nonattendance of their child as independent variables revealed that nonattendance of the child for dental appointments was significantly associated with three factors: activity temperament of the child (*p *= 0.019), lack of time (*p* = 0.041) and inconvenience (*p* < 0.001) as reasons for missed appointments. The Hosmer–Lemeshow test revealed that *χ*2 = 5.08 and *p* = 0.749, indicating a good fit of the model. Compared with attenders, nonattenders were likely to have higher activity temperament scores, with an odds ratio of 1.9. The parents of the nonattenders were 4 times and 16 times more likely to have a lack of time and inconvenience, respectively, as reasons for missing appointments (Table 8).

**Table 8:** Multiple logistic regression analysis to analyse the associations of dental attendance with the oral health literacy of the parent and the temperament of the child, along with demographic variables, time interval between appointments, and reasons for nonattendance

Variable		Wald	Odds ratio	CI	*P* value
				Lower–upper	
Age (child)		1.31	0.57	0.21–1.50	0.253
Sex (child)	Female (ref)	0.39	1.49	0.43–5.21	0.532
	Male				
Age (parent)		0.01	1.00	0.89–1.14	0.944
Sex (parent)	Female (ref)	2.50	0.32	0.08–1.32	0.114
	Male				
Socioeconomic status	Upper middle (ref)	0.16	0.66	0.09–5.09	0.690
	Upper Lower				
Oral health literacy	Adequate (ref)				
	Marginal	0.08	1.28	0.24–6.81	0.766
	Inadequate	0.51	0.34	0.02–6.56	0.474
Temperament	Emotionality	0.79	0.84	0.58–1.23	0.373
	Activity	5.49	1.90	0.11–3.25	0.019*
	Shyness	0.28	1.11	0.76–1.63	0.596
Interval between appointments		1.24	0.92	0.80–1.06	0.266
Inconvenience	No (ref)	14.82	16.00	3.90–65.62	< 0.001*
	Yes				
Lack of time	No (ref)	4.20	4.24	1.06–16.89	0.041*
	Yes				
Lack of availability of dentists	No (ref)	0.01	1.05	0.03–37.41	0.981
	Yes				
Fear of infectious diseases	No (ref)	1.05	6.03	0.19–186.89	0.305
	Yes				
Break in school attendance	No (ref)	0.16	1.65	0.14–19.73	0.691
	Yes				

^*^P < 0.05 = significant

## Discussion

The current cross-sectional study investigated the possible role of parental oral health literacy and the child's temperament in missed dental appointments in children with ECC. The study also included covariates such as demographic factors, parental attitudes and barriers to dental care utilisation. When controlling for the covariates, the activity temperament of the child was associated with dental attendance. Covariates such as inconvenience and lack of time as reasons for missed appointments were also associated with nonattendance.

In our study sample, 53% had ≥ 20% missed/cancelled appointments over the 6-month study period. The level of nonattendance is comparable to findings from previous research on dental attendance among children in India (Tandon et al. 2016; Bhatia et al. 2018). While Tandon et al. noted approximately 40% missed appointments among children under 15 years of age, Bhatia et al. reported 52% missed appointments for children between 1 and 18 years of age. The child’s illness, breaks in school attendance, parents'forgetfulness regarding the appointment, and parents'commitments to their work were the reasons for missed appointments in the abovementioned Indian studies. The proportion of missed dental appointments in our study was much greater than that reported from Nordic countries, which was 11%, possibly because of the free access to dental care provided in these countries (Gustafsson et al. 2010, 2018). Even in these countries, low socioeconomic status is associated with low dental attendance (Gustafsson et al. 2018). Similarly, in our study sample, which consisted mainly of middle- and low-socioeconomic-status participants, a high proportion were nonattenders. In contrast to our study results, Bhatia et al. (2018) reported more missed appointments in the high-income group in their study conducted at Navi Mumbai, India. The results of our study, following those of previous studies, revealed no associations between the age or sex of parents or children and dental attendance (Wang and Aspelund 2009; Bhatia et al. 2018). Earlier studies have reported that forgetfulness could be one of the most important factors for missed dental appointments (Skaret et al. 2000; Bhatia et al. 2018). The parents in our study received at least one telephone reminder call as part of the routine scheduling system. Hence, we did not include'forgot appointment'as a reason for missed appointments. Telephonic reminder calls substantially increase the patient show rate for recall appointments (Marbouh et al. 2020). Despite the reminder call, two parents expressed that they had forgotten the appointment, which was the reason for the inconvenience leading to nonattendance.

Approximately 74% of nonattenders reported ‘inconvenience’, such as ill health and family matters, as the reasons for missed appointments. ‘Inconvenience’ as a reason for nonattendance was substantially greater than those reported by the attenders. Parents of nonattenders are burdened with everyday life problems such as financial, psychosocial, physical, or mental health-related problems and may require support to cope with everyday life. As a result, parents may ignore dental treatment needs that do not require immediate attention (Hallberg et al. 2008; Gustafsson et al. 2010). The parents’ lack of time as a reason for missing dental appointments also reflects the low prioritisation of dentist appointments amid everyday life needs (Bhatia et al. 2018), which is evident from our study data, as all the children in the study sample attended our dental clinic only when they were in pain. Earlier studies have also shown that children with high dental caries experience visit their dentists only when pain arises (Gustafsson et al. 2010; Goettems et al. 2012). Children with high dental caries experience also have a greater burden of dental treatment. The regularity of dental visits may be influenced by the cost of dental services, household income, and time required to access dental services (Badri et al. 2014). According to the ‘accumulation of risk model’ concept, common risk factors, such as lower SES and factors affecting dental care accessibility, can affect both dental caries disease levels and the regularity of dental attendance (Mohd. Khairuddin et al. 2024).

The parents who participated in this study were aware of their child's tooth decay and the importance of maintaining their child's oral health, and they overrated their child's oral health as ‘good’ or ‘fair’. Thus, despite their awareness of their children's dental health care needs, they perceived their children's general and oral health as good and missed their children's dental appointments. The perception of poor oral health and parents'perceived need for dental care are important factors influencing their children's utilisation of dental care (Gustafsson et al. 2018; Al Agili and Farsi 2020). According to the health belief model, despite the awareness of their children's susceptibility to and severity of the disease, the parents of nonattenders in our study failed to realise that the benefits of continued dental treatment were greater than the barriers to dental attendance (Brega et al. 2020).

Missing appointments contribute to persistent increased dental caries experience levels in children (Hallberg et al. 2008; Wang and Aspelund 2009). Although unmet dental treatment needs are high among preschool children, dental care utilisation remains low (Camerini et al. 2020). The regularity of dental attendance represents dental service utilisation (Badran et al. 2023). The regularity of children's dental visits can be affected by parents'perceptions of the treatment needs of their children, whether untreated carious lesions affect their children's quality of life, or complaints of pain or swelling due to carious teeth (Sruthy et al. 2023). Hence, the questionnaire administered to parents included questions about parental perceptions of oral health and reasons for dental visits. The questionnaire was based on Anderson's PEN (predisposing, enabling and need-based) behaviour model, which explains dental service utilisation (Al Agili and Farsi 2020; Gao et al. 2020). We included predisposing factors (age, sex, and socioeconomic status), enabling factors (availability of dentists nearby, cost of dental treatment) and self-perceived need for dental treatment of their children, following the Anderson model.

According to our study, parental OHL was not associated with dental attendance. Most participating parents had adequate oral health literacy, with only 31 showing inadequate OHL. Among the parents with inadequate OHL levels, 17 (approximately 55%) were nonattenders. There has been conflicting evidence regarding the association of oral health literacy with the frequency of dental visits, with some studies showing a significant association (Shin et al. 2014; Menoncin et al. 2023; Ferreira et al. 2023; Gudipaneni et al. 2024), whereas others have shown no significant association (Divaris et al. 2014; Burgette et al. 2016; Yazdani et al. 2018). However, all these studies relied on the parent-reported frequency of dental visits in the last six months or one year. In a systematic review, since the methodological quality of the studies was low, the association of parental OHL with the frequency of dental visits could not be confirmed (Firmino et al. 2018). The significant association in earlier studies was attributed to low parental knowledge, resulting in an inability to follow instructions from the dentist (Naghibi Sistani et al. 2014; Al Agili and Farsi 2020). The association of parental OHL with dental attendance could not be established in our study; the difference in the results of the comparison could be due to differences in the population type and the type of instrument used (Alzeer et al 2024). We used the OHL-AQ questionnaire, as it is short, easy to use and includes components related to listening, decision-making, interpretation of prescriptions and comprehension of health information (Dickson-Swift et al. 2014; Naghibi Sistani et al. 2014). The other existing instruments measure only limited dimensions of oral health literacy, such as knowledge and reading comprehension (Firmino et al. 2018; Jangid et al. 2024). Hence, tools that include broader concepts such as behaviour, decision-making, service navigation, and are sensitive to local contexts and languages are needed (Dickson-Swift et al. 2014; Alzahrani et al. 2024).

Our findings suggest that parents'OHL may not affect consistent dental care utilisation by children with ECC. However, the results should be interpreted within the limitations of the study sample. The participating parents belonged to the middle SES group, had a favourable attitude toward their child's oral health and understood that dental therapy was necessary. However, they visit the dentist only when a complaint of pain arises from their child, with a lack of time and inconvenience being the most common reasons for inconsistency in attending dental appointments. The study sample included parents of children visiting the dental clinic for treatment, whose OHL may differ from that of parents of children who never visited the dental clinic for treatment. Dental visits can increase parents'knowledge of oral health and OHL (Mallineni et al. 2023; Alzahrani et al. 2024). Our study may be considered an exploratory study on the association of parental OHL with the dental attendance of children for scheduled recall appointments. Thus, further studies on Indian populations with different SES levels may be needed. Furthermore, the influence of parental OHL on other preventive behaviours, such as tooth brushing and avoiding sugar intake, in the Indian population needs to be investigated, as children who lack regularity in dental attendance are also likely to have poor tooth brushing habits and greater sugar exposure in their diet (Leroy et al. 2013; Alhareky and Nazir 2021).

We used the EAS scale, a modified version of the EASI (emotionality, activity, sociability and impulsivity) scale, for the measurement of temperament in this study, as it has been used extensively, with reliability and validity measured, rendering it suitable for use across cultures (Ohashi and Kitamura 2017). It has shown stability with time in a longitudinal study, making it a suitable measure for use as a predictor variable (Bould et al. 2013). We used a three-factor scale, as the impulsivity and sociability temperament traits overlapped in earlier studies with activity and shyness temperaments, respectively (Gasman et al. 2002; Ohashi and Kitamura 2019). The EAS represents the most heritable personality trait and is valid across all ages (Boer and Westenberg 1994).

Activity temperament was associated with nonattendance among the three temperaments measured via the EAS scale. The activity temperament has been associated with crying behaviour in five-year-olds (Radis et al. 1994). Children with increased mean activity temperaments tend to have increased dental fear scores and dental behaviour management problems (Klingberg and Broberg 1998; Juárez-López et al. 2022). Owing to their impulsive temperament related to their activity temperament, children may lack patience and perseverance (Arnrup et al. 2007). As a result, children have difficulty devising problem-focused coping strategies in stressful situations such as dental treatment, which leads to behaviour management problems, which can be one of the reasons for missed dental appointments (Arnrup et al. 2007; Gustafsson et al. 2010). In a recent study, high activity scores were associated with Frankl’s negative and definitely negative behaviour (Juárez-López et al. 2022). A lack of cooperation by a child can influence parental motivation and responsibility to bring their children to dental appointments (Arnrup et al. 2002). As these children may have difficulty staying still during dental examination and treatment, the use of appropriate behaviour guidance strategies by the paediatric dentist, such as distraction, positive reinforcement in the form of praise/rewards and providing breaks, may be helpful (Juárez-López et al. 2022).

This study has several limitations. Data from a short period of 6 months were gathered with a small sample size and from a single paediatric clinic of a dental school. Hence, the nonattendance behaviour observed in this study may not be representative of the population in the area. However, the data on missed appointments were gathered from the dental records rather than the parents, as in earlier studies (Gustafsson et al. 2018; Bhatia et al. 2018), thus making the dental attendance data of our study more objective and realistic. We assessed the test‒retest reliability of the questionnaire for the study sample, which was excellent. As several factors can contribute to nonattendance, we included other covariates that can influence nonattendance behaviour. Although we aimed to make the questionnaire comprehensive by addressing multiple criteria, several other factors may have contributed to nonattendance, such as parental anxiety, the child's dental caries status, past dental behaviour and other psychosocial characteristics. The parents'response to the questionnaire could have been influenced by social desirability, a limitation of the cross-sectional design. Owing to the cross-sectional design of our study, only an association of the significant risk factors can be attributed to nonattendance, and causality cannot be confirmed.

Understanding the risk factors for nonattendance of dental appointments will aid in developing awareness and motivational programs for parents of children with ECC, resulting in better utilisation of dental health services for their children. Our study results help define the possible associations of two possible risk factors for nonattendance, child temperament and parental oral health literacy, along with common reasons provided by parents for nonattendance. Further research on the role of parental OHL and children's temperaments in other populations is needed. Studies conducted with case‒control or cohort designs can confirm whether oral health literacy and temperament are predictors of nonattendance for scheduled dental appointments among children with ECC.

## Conclusion

Within the limitations of this study, nonattendance for dental appointments among children with ECC was not associated with parental OHL. The temperament of the child’s activity, lack of time and parents’ perceptions that dental appointments are inconvenient are associated with nonattendance among children with ECC.

## Data Availability

The data that support the findings of this study are available from the corresponding author upon reasonable request.
